# Roles of IL-34 in neurological diseases: neuroprotection, inflammatory regulation, and myeloid plasticity

**DOI:** 10.3389/fimmu.2026.1898176

**Published:** 2026-08-20

**Authors:** Yilong Peng, Chenyang Jin, Yuewen Sun, Wenfeng Mu, Xueping Zheng

**Affiliations:** 1 Department of Geriatric Medicine, The Affiliated Hospital of Qingdao University, Qingdao, Shandong, China; 2 Department of Clinical Laboratory, Qingdao Women and Children's Hospital, Qingdao, Shandong, China

**Keywords:** Alzheimer’s disease (AD), colony-stimulating factor-1 receptor (CSF-1R), immunometabolism reprogramming, interleukin-34 (IL-34), neurodegenerative diseases

## Abstract

Interleukin-34 (IL-34) is a brain-enriched cytokine and a tissue-restricted ligand of colony-stimulating factor-1 receptor (CSF-1R) that plays an important role in microglial development, survival, and functional specialization. A growing body of evidence indicates that IL-34 is dysregulated in both central and peripheral nervous system diseases and exerts pleiotropic effects not only through CSF-1R but also through non-canonical receptors, including TREM2, syndecan-1, and PTP-ζ. Under physiological conditions, IL-34 is constitutively produced predominantly by neurons across both the central and peripheral nervous systems. However, in pathological settings, its cellular sources expand dynamically: in CNS diseases, IL-34 is primarily derived from stressed neurons and reactive astrocytes, whereas in PNS disorders, it is robustly upregulated by injured sensory neurons, satellite glial cells, and reactive Schwann cells. IL-34 contributes to the regulation of resident glia in the CNS, while evidence from peripheral immune disorders suggests that it may also influence infiltrating myeloid-cell adaptation under pathological conditions. In this review, we highlight the multiple effects of IL-34 and its receptor network on CNS-resident, peripheral glial, and peripheral immune cells, with particular attention to the available data supporting its dual role in neuroprotection and inflammatory amplification. We synthesize recent findings regarding IL-34’s role in myeloid reprogramming, particularly in orchestrating transcriptional and functional phenotypic shifts. While current literature provides evidence linking IL-34 to these transcriptional changes, its contribution to metabolic and epigenetic remodeling remains an emerging field. Furthermore, our findings summarized in this manuscript may help evaluate whether targeting IL-34-related pathways can be exploited for therapeutic intervention in neurodevelopmental, neurodegenerative, cerebrovascular, and peripheral nerve pathologies.

## 1 Introduction

IL-34 was discovered in 2008 as the second ligand for the colony-stimulating factor-1 receptor (CSF-1R). Before IL-34 was discovered, colony-stimulating factor-1 (CSF-1) was thought to be the only ligand for CSF-1R (1). In comparison to CSF-1, the expression of IL-34 demonstrates significant tissue specificity. Preliminary analysis of mRNA revealed that the level of IL-34 is particularly elevated in the brain and skin, while its expression levels are comparatively low in other tissues, including the liver, spleen, kidneys, thymus, testis, and others (2). Further studies at the tissue and cellular levels have shown that in the central nervous system (CNS), IL-34 is mainly produced by neurons in regions such as the cerebral cortex, hippocampus, and striatum, and plays an important role in maintaining the number and homeostasis of local microglia (3). In the skin, keratinocyte-derived IL-34 is the principal trophic cue supporting the Langerhans cell pool via CSF-1R activation (4). By contrast, IL-34 expression is comparatively modest in peripheral organs such as the liver and intestine; nevertheless, available evidence suggests that it participates in sustaining local macrophage survival and shaping their tissue-adapted functions (5).

CSF-1R is an important receptor for myeloid lineage cells. In the periphery, it is mainly expressed in the monocyte-macrophage system, including monocytes, tissue macrophages, bone marrow progenitor cells, dendritic cell subsets, and osteoclasts. In the CNS, it is almost exclusively expressed in microglia (6). Although both IL-34 and CSF-1 exert their effects through the CSF-1R receptor, several biophysical studies have shown that IL-34 generally binds CSF-1R with equal or slightly higher affinity compared with CSF-1. Using surface plasmon resonance, researchers first showed that IL-34 has a slower dissociation rate from CSF-1R, resulting in a lower apparent equilibrium dissociation constant (KD) than CSF-1 under comparable conditions (7). This difference in affinity is believed to lead to changes in the downstream signaling kinetics and the biased activation of CSF-1R. This results in different ligands acting on the same receptor, but there are still differences in the microglial cell phenotypes in specific regions of the CNS. The reprogramming function and inflammatory amplification function of IL-34 have been studied in greater depth in different disease contexts. The proliferation and activation of microglia are hallmarks of neurodegenerative diseases such as Alzheimer’s disease (AD), Parkinson’s disease (PD), and cerebral small vessel disease (CSVD). Therefore, strategies to regulate microglia by blocking IL-34 or CSF-1R are currently being evaluated in order to treat neurodegenerative diseases. This review will explore the functions of IL-34 and its connections with neurological disorders from a physiological to a pathological perspective, and from the central nervous system to the peripheral nervous system.

## 2 The biological characteristics and receptors of IL-34

### 2.1 The gene location and structure of IL-34

IL-34 is encoded by the IL34 gene on human chromosome 16q22.1 and is translated as a 222-amino-acid precursor (8). Structurally, IL-34 is a secreted glycoprotein that assembles into a non-covalent homodimer and adopts a four-helix bundle type cytokine fold. Notably, its dimeric architecture is often discussed in the context of its structural similarity to cystine-knot growth factors, such as PDGF and VEGF. Under physiological conditions, IL-34 displays substantial extracellular stability; post-translational glycosylation is thought to support proper folding and to extend ligand persistence, thereby favoring sustained receptor engagement in tissue niches (9). Collectively, these biochemical features position IL-34 as a durable CSF-1R ligand capable of maintaining long-range signaling competence in extracellular environments.

### 2.2 IL-34 and its receptors

#### 2.2.1 IL-34 and CSF-1R

Under physiological conditions, CSF-1R represents the dominant IL-34 signaling axis in CNS-resident microglia. CSF-1R is the principal signaling receptor for IL-34 and is indispensable for the maintenance of CNS myeloid homeostasis, particularly the survival and functional fitness of microglia. Monoallelic loss-of-function mutations in CSF-1R result in adult-onset leukoencephalopathy with axonal spheroids and pigmented glia (ALSP), a hereditary neurodegenerative disorder that clinically resembles Alzheimer-like dementia and is frequently accompanied by progressive motor impairment and seizures (10). At the molecular level, IL-34 binding promotes CSF-1R dimerization and receptor autophosphorylation, triggering intracellular signaling programs that include JAK-STAT, PI3K-Akt, MAPK pathways such as ERK1/2 and JNK, and NF-κB. These cascades coordinate key myeloid cell behaviors, including proliferation, survival, differentiation, migration, and cytokine production, thereby shaping microglial maintenance and responsiveness (11). Beyond its effects on resident microglia, CSF-1R signaling also influences the differentiation and function of monocyte-derived macrophages that enter the CNS during disease. A recent study showed that IL-34/CSF-1R signaling and CCR2-dependent recruitment tune the abundance and polarization of Myeloid-Derived Suppressor Cells (MDSCs), providing potential levers to modulate engrafted populations (12). Furthermore, under pathological conditions such as stroke, traumatic brain injury, and neuroinflammation, large numbers of peripherally derived monocyte-derived macrophages (MDMs) infiltrate the brain parenchyma and exhibit phenotypic and functional properties distinct from microglia, suggesting that peripheral myeloid cells may significantly contribute to the pathogenesis of neurodegenerative diseases (13). Although CSF-1R has been the focus of multiple therapeutic strategies, its broad expression in both central and peripheral tissues poses challenges for selectively modulating CNS immunity. Targeting IL-34, which has a more restricted expression pattern in the CNS, may offer a more specific approach while minimizing unintended effects on peripheral myeloid populations. However, effective delivery of IL-34 targeting agents to the brain remains limited by the blood-brain barrier (BBB), and therefore optimized intracerebral or CNS selective delivery strategies require further investigation.

#### 2.2.2 IL-34 and TREM2

Triggering receptor expressed on myeloid cells-2 (TREM2) functions as a major immunoregulatory hub in myeloid cells. TREM2-dependent IL-34 signaling appears to emerge primarily under conditions associated with cellular stress, lipid accumulation, and tissue damage. In physiological settings, its expression is enriched in tissue-resident compartments, including CNS microglia, consistent with a role in maintaining homeostatic programs. Under pathological conditions, ligands released or exposed in damaged tissue activate TREM2 signaling, thereby coupling danger sensing to phagocytic, metabolic, and inflammatory responses that can restrain tissue injury and prevent secondary spread (14). Upon ligand engagement in injured tissue, TREM2 signals through the adaptor DAP12 (TYROBP). Phosphorylation of the DAP12 immunoreceptor tyrosine-based activation motif creates a docking platform for SYK, which then propagates downstream PI3K, AKT, and mTOR signaling. Together, these pathways coordinate microglial phagocytosis, lipid metabolism, metabolic rewiring, and survival, and facilitate the acquisition of the disease-associated microglia (DAM) phenotype, an injury-responsive state implicated in both damage containment and neurodegenerative disease progression (15, 16). In the CNS, TREM2 links damage perception to the remodeling of adaptive microglia.

Recent research has identified TREM2 as a novel receptor for IL-34, expanding the functional scope of IL-34 beyond its canonical interaction with CSF-1R. In myeloid malignancies, IL-34/TREM2 signaling appears to exert strong pro-differentiation effects. Tsc1-deficient osteoclasts secrete elevated levels of IL-34, which drive acute myeloid leukemia (AML) cell differentiation and restrain disease progression *in vivo*. Consistently, IL-34 promotes the differentiation of primary murine leukemia cells and human AML cell lines through TREM2 engagement, and this effect is recapitulated in xenograft models established with primary human AML cells. Mechanistically, IL-34 directly binds TREM2, induces rapid phosphorylation of Rasal3, and suppresses ERK1/2 signaling, thereby favoring leukemic cell differentiation (17). Importantly, evidence supporting IL-34-mediated myeloid reprogramming derives from heterogeneous experimental systems, including peripheral inflammatory diseases, hematological malignancies, animal models, and human-derived cellular systems. The extent to which these mechanisms operate in CNS-resident microglia requires further validation. Within the CNS, IL-34-TREM2 signaling has so far been characterized primarily in AD. Neuron-derived IL-34 engages TREM2 on microglia and exerts neuroprotective effects by attenuating the NLRP3 inflammasome activation and reducing the production of pro-inflammatory cytokines (18). Beyond AD, however, the functional consequences of IL-34 acting as a putative non-canonical TREM2 ligand remain undefined. Whether this pathway influences microglial transition toward the DAM phenotype, metabolic reprogramming, or phagocytic behaviour in other neurological disorders remains an open question that warrants systematic investigation.

#### 2.2.3 IL-34 and SDC-1(CD138)

In contrast, Syndecan-1 (SDC-1) may function primarily as a context-dependent co-receptor rather than an independent signaling receptor. SDC-1 has recently been identified as a co-receptor for IL-34, adding a new layer of complexity to IL-34-mediated signaling in peripheral immune metabolism and CNS pathology. SDC-1 is a transmembrane heparan sulfate proteoglycan predominantly positioned on the basolateral surface of epithelial cells. It consists of a core protein decorated with heparan sulfate and chondroitin sulfate glycosaminoglycan chains, which enable it to bind diverse ligands and function as a versatile organizer of cell-matrix and cell-cytokine interactions (19). SDC-1, a type I transmembrane heparan sulfate proteoglycan, participates in a broad spectrum of fundamental cellular processes, including cell adhesion, extracellular matrix interactions, cell cycle progression, and lipid metabolism. By engaging a diverse repertoire of growth factors, cytokines, and matrix components, SDC-1 modulates key signaling pathways, including MAPK, PI3K/AKT, NF-κB, and Wnt/β-catenin, and thereby shapes inflammatory responses, tissue remodeling, and tumor progression (20, 21). Within the CNS, epithelial cells of the choroid plexus represent the predominant site of SDC-1 expression (22). Elevated SDC-1 expression is also observed in developmental germinal zones, including retinal ganglion cell precursors and intermediate progenitor cells, whereas differentiated neurons in the developing cerebral cortex largely lack SDC-1, consistent with a preferential association with neural progenitor populations (23). In neuroinflammatory disorders such as multiple sclerosis(MS), increased cerebrospinal fluid CD138 has been proposed as a disease-associated biomarker, and experimental evidence further suggests selective activation of a myelin basic protein 3-like protein 1/CD138 signaling axis in oligodendrocyte lineage cells (24). In addition, SDC-1 is increasingly recognized as a marker of endothelial injury and coagulation abnormalities after traumatic brain injury, as serum SDC-1 concentrations rise in patients with a single traumatic event and increase further following repeated insults (25). Nevertheless, the contribution of SDC-1 to the regulation of neuroinflammation in the CNS remains incompletely defined, and its robustness as a biomarker continues to be debated.

A study conducted Scatchard analysis and inhibition experiments and utilized surface plasmon resonance technology to investigate the binding situation of IL-34. It was found that, apart from CSF-1R and PTP-ζ, IL-34 also has a low affinity binding with the chondroitin sulfate chain. In proteoglycans containing chondroitin sulfate chains, SDC-1 can regulate the CSF-1R signaling pathway induced by IL-34. Moreover, IL-34 can induce the migration of cells expressing SDC-1 (26). In the brain metastases of lung adenocarcinoma, the expressions of IL-34, its receptor CSF-1R, and SDC-1 are significantly increased, which also suggests that IL-34 plays an important role in the migration process (27). Furthermore, SDC-1 functions as a crucial co-receptor that regulates the IL-34/CSF-1R pathway, establishing a mechanistic link between IL-34-mediated signaling and immunometabolic reprogramming in peripheral inflammatory diseases. In rheumatoid arthritis (RA), SDC-1-mediated cooperative signaling enhances CSF-1R phosphorylation, which shifts pro-inflammatory macrophages toward glycolysis via upregulating GLUT1, HIF-1α, and c-MYC, ultimately intensifying synovial inflammation and bone erosion through cytokine production and osteoclastogenesis damage (28). However, studies on this axis are only limited to peripheral inflammation. Whether pathological stimulation can induce the expression of SDC-1 in microglia and whether this axis also regulates the recruitment and metabolic reprogramming of infiltrating immune cells in the brain are still uncertain, which may be potential research problems in neuroinflammation in the future.

#### 2.2.4 IL-34 and PTP-ζ

PTP-ζ represents another non-classical IL-34 receptor with distinct cellular distribution and biological functions. Unlike CSF-1R and TREM2, which are primarily associated with myeloid regulation, PTP-ζ is expressed in neural progenitor cells, neurons, and glial populations and appears to regulate cell adhesion, migration, and developmental processes (29). By IL-34 affinity chromatography of solubilized mouse brain membrane followed by mass spectrometric analysis, PTP-ζ was identified as a novel IL-34 receptor (30). The interaction between IL-34 and PTP-ζ can induce a series of signaling cascades that inhibit motility, clonogenicity, and proliferation of specific cell types, via tyrosine phosphorylation of paxillin and focal adhesion kinase (FAK) (31). Studies have shown that treatment with anti-IL-34 antibodies can inhibit the intrarenal expression of IL-34 and PTP-ζ. Moreover, by inhibiting macrophage infiltration, it can alleviate interstitial damage in the renal tubules and significantly improve renal function indirectly (32). PTP-ζ is regarded as a non-classical/auxiliary receptor of IL-34. The signaling pathway and biological functions remain unclear.

IL-34 engages multiple receptors, and this versatility is central to its context-dependent functions in the CNS. Under homeostatic conditions, microglia predominantly utilize CSF-1R, through which IL-34 supports their survival, differentiation, and surveillance activities, thereby maintaining myeloid equilibrium. In pathological settings, however, altered receptor expression and ligand presentation enable IL-34 to signal through additional receptors. Upregulation of TREM2 can divert IL-34 signaling toward pathways that promote metabolic adaptation, phagocytic competence, and controlled inflammasome activity, contributing to protective injury responses. Conversely, SDC-1 may enhance CSF-1R signaling under inflammatory conditions, potentially driving glycolytic reprogramming and maladaptive activation of microglia or infiltrating myeloid cells. PTP-ζ, expressed in neural progenitors, neurons, and glia, appears to mediate adhesion and migration-related responses, though its role in CNS pathology remains poorly defined. Overall, receptor availability, cellular phenotype, glycosaminoglycan-dependent ligand presentation, and the inflammatory milieu collectively determine IL-34’s signaling preference and whether its effects are protective or detrimental.

In conclusion, in physiological conditions, CSF-1R is the predominant IL-34 receptor in microglia and mediates essential functions including survival, proliferation, and maintenance of microglial homeostasis. However, under pathological conditions such as neurodegeneration, chronic inflammation, or tissue injury, changes in receptor expression and cellular state may alter IL-34 responsiveness. TREM2-dependent IL-34 signaling represents an additional regulatory pathway, particularly in activated or disease-associated microglial states, where it may influence phagocytosis, lipid metabolism, and metabolic adaptation. Thus, in diseases such as AD, CSF-1R and TREM2 signaling should not be considered opposing pathways; rather, their relative activation and interaction determine whether IL-34 promotes effective microglial adaptation or contributes to dysfunctional inflammatory responses.

**Figure 1:**
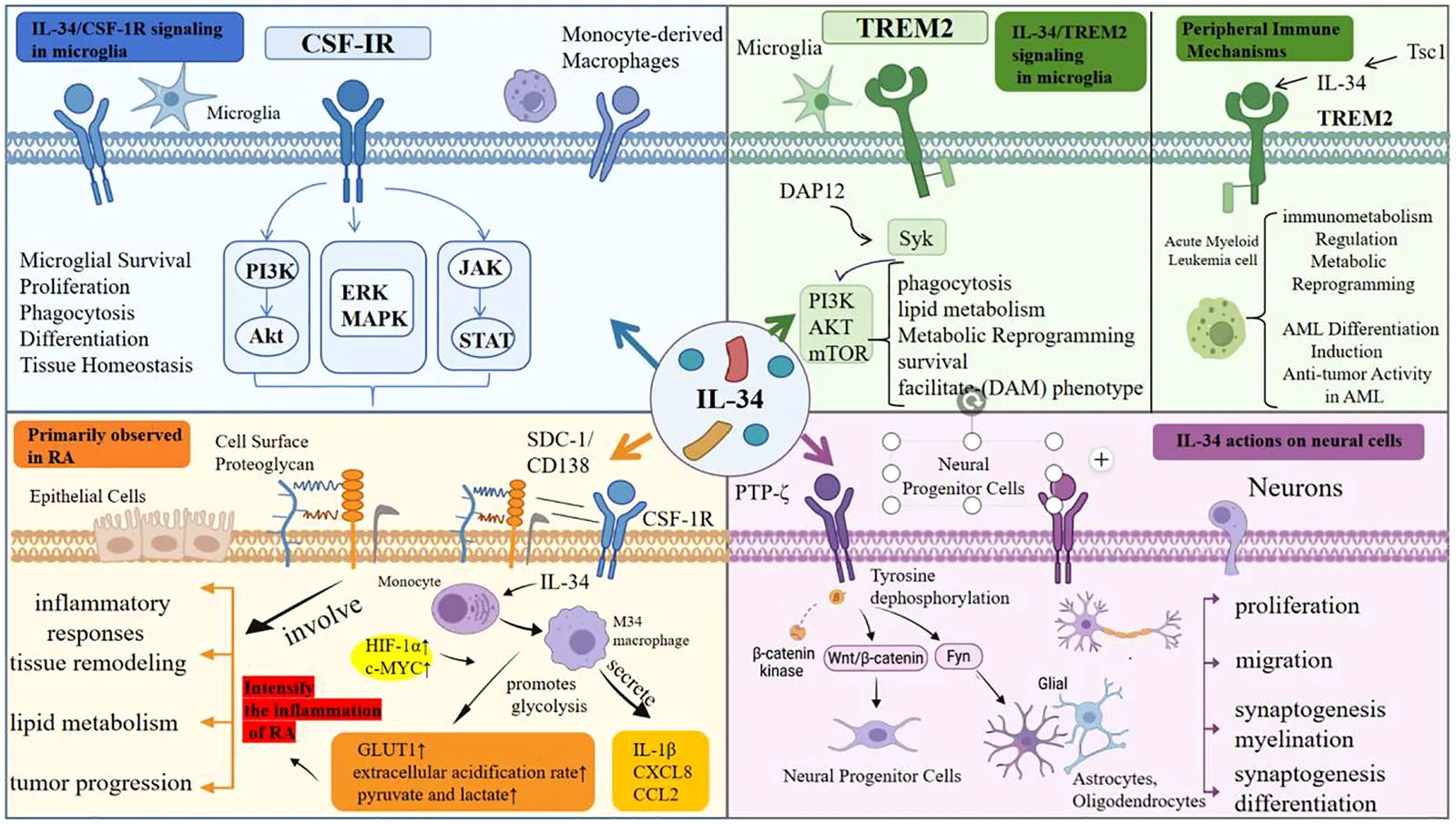
IL-34 and its receptors: differential signaling pathway in the CNS and peripheral immunity. In the CNS, IL-34 regulates microglial homeostasis and neuroimmune responses mainly through CSF-1R and TREM2, while also influencing neural progenitor cells and neuronal development via PTP-ζ. Outside the CNS, representative examples from rheumatoid arthritis (RA) and acute myeloid leukemia (AML) illustrate the broader immunometabolic functions of IL-34 in peripheral immune cells. These peripheral mechanisms are included to provide biological context and are not the primary focus of this review.

The non-classical receptors SDC-1 and PTP-ζ further expand the functional diversity of IL-34 signaling. SDC-1 may regulate IL-34 availability and receptor presentation through glycosaminoglycan-dependent mechanisms, thereby modifying CSF-1R-associated responses in inflammatory environments. PTP-ζ, which is expressed in neural and glial populations, may mediate IL-34 functions related to cell adhesion, migration, and neural development. Collectively, IL-34 should be viewed as a context-dependent signaling molecule whose functional outcome is determined by receptor distribution, cellular state, and disease-specific microenvironments (**Figure 1**).

## 3 IL-34 within inflammatory cytokine networks

Current evidence indicates that IL-34 participates in complex inflammatory networks in which its expression and biological activity are dynamically regulated by surrounding cytokines. Rather than functioning as a strictly upstream regulator, IL-34 both responds to inflammatory stimuli and, once induced, modulates downstream immune-cell activation through CSF-1R-dependent signaling. Consequently, many reported associations between IL-34 and inflammatory mediators reflect bidirectional feedback interactions rather than simple cause-and-effect relationships. Distinguishing correlation from causality is therefore essential when interpreting the role of IL-34 in inflammatory diseases.

In pemphigus lesions, for example, activated keratinocytes produce IL-34, yet this expression is itself stimulated by cytokines such as TNF-α. IL-34, together with TNF-α, can promote the differentiation of infiltrating monocytes into IL-1β- producing inflammatory macrophages, a process associated with enhanced chemotactic activity. IL-34 can also induce Th17-related mediators, including CCL20, IL-6, and IL23A (33, 34), however, these observations mainly indicate associations within an inflammatory milieu, and do not establish whether IL-34 is the initiating factor or a downstream responder to pre-existing cytokine activation.

Similar bidirectional interactions have been reported in periodontitis. TNF-α and IL-1β induce IL-34 expression in gingival fibroblasts, whereas IL-34 subsequently promotes RANKL-dependent osteoclastogenesis by functionally substituting for CSF-1 (35). These data support IL-34’s involvement in inflammatory and osteoclastogenic processes.

Within the CNS, available evidence likewise supports reciprocal regulation rather than a unidirectional signaling hierarchy. Microglia and infiltrating monocytes represent both targets and sources of inflammatory cytokines, and IL-34-mediated CSF-1R signaling is integrated into this broader cytokine network. Experimental evidence further indicates that IL-34 alone is insufficient to robustly induce IL-1β, CCL20, or other inflammatory mediators in microglia. Instead, IL-34 generally requires concurrent inflammatory signals—including TNF-α, IL-1β, or damage-associated molecular patterns (DAMPs)—to elicit full pro-inflammatory activation. Conversely, these cytokines also increase IL-34 expression or enhance CSF-1R responsiveness, thereby establishing reciprocal feed-forward interactions during chronic neuroinflammation (36). Overall, available evidence does not support a simple linear model in which IL-34 uniformly regulates other inflammatory mediators. Rather, IL-34 should be considered a context-dependent component of dynamic cytokine networks that both responds to and modulates inflammatory signaling. Whether IL-34 functions predominantly as a downstream responder, an upstream amplifier, or a homeostatic regulator depends on the tissue context, the composition of the cytokine milieu, and the activation state of responding myeloid cells. Therefore, many reported associations between IL-34 and inflammatory mediators should be interpreted as reciprocal regulatory interactions rather than direct causal relationships.

## 4 The expression patterns and functions of IL-34 in the CNS

IL-34 and CSF-1 are markedly different in their temporal expression, regional distribution, and cellular origin, resulting in non-redundant functions in the CNS. During embryonic and early postnatal development, CSF-1 is broadly expressed across multiple brain regions and is initially produced mainly by microglia, later also by oligodendrocytes and astrocytes. By contrast, IL-34 expression is more restricted and enriched in forebrain structures, including the cortex and hippocampal-related areas, and is predominantly derived from neurons, particularly excitatory neurons in developing forebrain circuits.

One developmental study illustrates that the level of IL-34 increases during the second postnatal week in forebrain regions such as the anterior cingulate cortex, coinciding with a critical period of synaptic refinement. Genetic or antibody-mediated loss of IL-34 reduces microglial density and the expression of homeostatic markers such as TMEM119, while increasing lysosomal marker CD68 and aberrant synaptic engulfment (11). In a chronic neurodegeneration model, Obst et al. showed that IL-34 is a tissue-restricted CSF-1R ligand that is highly enriched in the brain, supports microglial maintenance and proliferation, yet is largely dispensable for most peripheral macrophage populations. Systemic IL-34 blockade spared circulating monocytes and liver–kidney macrophages but reduced skin Langerhans cells, in contrast to broad CSF-1R inhibition, which depleted multiple myeloid compartments (37). These observations indicate that IL-34 has functions as a neuron-derived regulatory signal that restrains excessive microglial synaptic pruning and supports proper cortical circuit development. Notably, neutralization of CSF-1 during the same developmental window does not reproduce the microglial defects seen with IL-34 blockade in the anterior cingulate cortex, further supporting a specialized role for IL-34 in microglia-neuron communication.

IL-34 and CSF-1 also differ in their contribution to BAM biology. Border-Associated Macrophages (BAMs) are specialized resident macrophages located at CNS interfaces such as the meninges, perivascular spaces, and choroid plexus, where they contribute to vascular regulation, matrix remodeling, and immune surveillance. Their dependence on CSF-1R signaling implies that local CSF-1R ligands are critical for their development and maintenance, but the relative contributions of CSF-1 and IL-34 have only recently begun to be defined. Van Hove et al. showed that IL-34, produced by perivascular niche cells, including perivascular fibroblasts and mural cells, is essential for the maintenance of BAMs in adulthood (38). IL-34 deficiency causes a marked loss of BAMs and is associated with altered cerebrovascular dynamics, including increased arterial flow velocity and elevated cerebral blood flow. These findings indicate that IL-34, through sustaining BAM homeostasis, indirectly supports cerebrovascular function at the brain periphery interface (39). In conclusion, IL-34 and CSF-1 share CSF-1R but preferentially engage distinct biological programs, CSF-1 provides broad trophic support for microglia across the CNS, whereas IL-34 is more closely linked to neuron-derived, region-specific regulation of microglial maturation, synaptic pruning, and circuit formation, as well as the maintenance of BAMs and cerebrovascular integrity. These differences underscore that IL-34 and CSF-1 activate overlapping yet functionally distinct CSF-1R-dependent pathways during brain development and homeostasis.

## 5 Microglia and macrophages in nervous system diseases

### 5.1 The role of microglia in neurodegenerative diseases

Microglia are the main immune cells in the brain parenchyma and their survival and differentiation are regulated by CSF-1R. During embryonic development, microglia and neurons appear simultaneously and actively participate in the formation of neural circuits by regulating the number of neurons (40, 41). Under normal physiological conditions, microglia conduct patrols within the brain microenvironment, offering immune surveillance and facilitating the survival of neurons (42, 43). Neurons and microglia communicate with each other through multiple genes with neuronal expression, including chemokine (C-X-C) ligand 1 (CXCL1), cerebrospinal fluid 1, interleukin 34 (IL-34), and transforming growth factor β1 (TGF-β1) (44, 45). Microglia are involved in brain development and its homeostasis because they participate in the programmed cell death of neurons during the developmental process (46, 47). One animal study (48) has shown that microglia regulate neural progenitor cells and neuronal populations by phagocytosing apoptotic or excess cells, thereby refining the neural network (49). Neurodegenerative diseases are characterized by the progressive aggregation of harmful proteins, neuronal loss and chronic neuroinflammation (50). Under pathological conditions, microglia are activated and aggregated at the site of nerve injury, accompanied by significant morphological and transcriptional changes. Microglia experience dynamic alterations, with their morphology transitioning from a branched state (when in a resting condition) to an amoeboid state (when in an active condition). These disease-specific phenotypes are collectively referred to as DAM (16).

#### 5.1.1 The role of microglia in AD

In AD, the animal studies have shown that the binding of Aβ to Toll-like receptor 4 (TLR4), one of the pattern recognition receptors(PRRs), is believed to activate microglia. The TLR4-mediated microglial signaling pathway is involved in regulating the increase of inflammatory cytokines, the inhibition of phagocytosis, and the increase of plaque deposition (51, 52). Moreover, Aβ peptides can also activate NLRs, especially NLRP3, by promoting the formation of inflammatory bodies complexes and the maturation of IL-1β, thereby triggering an inflammatory response (53).

The receptors TREM2 expressed on the surface of microglia, when activated, can promote the migration, phagocytosis, proliferation and survival of microglia (54–56). Common extracellular ligands of TREM2 include anionic lipids, APOE, Aβ, and apoptotic cells (57, 58). The latest research has discovered that IL-34 can also act as a ligand for TREM2 (17). In the TREM2 mutant, the interaction between TREM2 and its ligand is impaired, which may be one of the reasons for the increased risk of AD (59, 60). Moreover, when Aβinteracts with microglia expressing TREM2, it can activate downstream target genes, ultimately promoting the clearance of Aβ. From a mechanism perspective, the TREM2 signaling pathway can promote the survival of microglia, maintain their metabolic health status, and enhance their phagocytic clearance function. In summary, in the pathogenesis of AD, the activation of microglia may play a dual role. On the one hand, acute microglial activation reduces the accumulation of Aβ by increasing its phagocytic or clearance capacity. On the other hand, the long-term activation of microglia triggers multiple pro-inflammatory cascades, promoting neurotoxicity and synaptic loss.

#### 5.1.2 The role of microglia in Parkinson’s disease

PD is the second most common neurodegenerative disorder, and microglial cell activation is also one of the significant characteristics of PD (61). In PD, many changes occur within the substantia nigra-striatum projection area (SNpc) dopaminergic neurons, includingα-synuclein synthesis and release disorders, protein degradation problems, mitochondrial dysfunction, etc (62). Activated microglia are largely expressed in the SN of the post-mortem brains of patients with PD (63). Anatomical examination of the brain tissues of patients diagnosed with PD was conducted, particularly focusing on the SNpc and the basal ganglia. The analysis revealed that in the regions where microglia were activated, the density of nitroso-α-synuclein (N-α-syn) increased. Additionally, the analysis of NF-κB expression indicated that the expression level of NF-κB in the SNpc was elevated, especially more pronounced when stimulated by N-α-syn, which explains the involvement of NF-κB in the inflammatory response of PD (64). Furthermore, one study has shown that activated microglia can induce A1 astrocytes by secreting IL-1α, TNF and C1q. Furthermore, they showed that A1 astrocytes are involved in the death of neurons and oligodendrocytes in neurodegenerative diseases (65). These evidence indicate that the activation of microglia in PD tends to have a detrimental effect.

#### 5.1.3 The role of microglia in multiple sclerosis

In MS, the disease is characterized by neurodegeneration and inflammatory demyelination (66). Activated microglia can be observed both within active lesions and in the seemingly normal white matter. In these latter cases, the clusters of activated microglia are closely associated with axonal damage, oligodendrocyte degeneration, and complement activation, indicating that microglia are involved in diffuse pathological processes beyond focal lesions (67). Importantly, microglial responses in MS are highly dynamic and context-dependent, involving both protective and detrimental functional states.

During early demyelinating injury, microglia contribute to tissue repair by clearing myelin debris and supporting oligodendrocyte precursor cell (OPC) survival and differentiation. In experimental models, microglia release neurotrophic and pro-remyelinating factors, including IGF-1, BDNF, semaphorin 3F, transglutaminase, and activin-A, which promote OPC maturation and remyelination (68, 69). In addition, microglial phagocytosis mediated by receptors such as TREM2, MerTK, CD36, and CX3CR1 facilitates the removal of inhibitory myelin debris and creates a permissive environment for repair (70, 71). Consistent with these protective functions, CSF-1R-dependent microglial depletion during the chronic phase of experimental autoimmune encephalomyelitis (EAE) using PLX3397 resulted in enhanced CNS T-cell proliferation and exacerbated disease severity, suggesting that microglia may normally restrain excessive adaptive immune activation (72). However, interpretation of CSF-1R-targeting studies requires caution, as these approaches also affect peripheral monocytes, macrophages, and other CSF-1R-expressing populations.

Conversely, persistent microglial activation in chronic MS may promote neurodegeneration. Pharmacological inhibition of CSF-1R signaling or c-Fms kinase activity in EAE models reduced degeneration-associated microglial states, attenuated axonal damage, and improved clinical outcomes (73, 74). These findings suggest that prolonged microglial activation may shift from a reparative state toward inflammatory and neurotoxic programs. However, differences between experimental models and human MS—including age, disease duration, and the extent of microglial manipulation—limit direct translation. Complete microglial depletion achieved in animal studies does not reflect the therapeutic goal in patients, where restoring homeostatic and protective microglial functions is likely more desirable than eliminating microglia. Therefore, microglia in MS should be viewed as highly plastic immune regulators whose functional consequences depend on disease stage, inflammatory environment, and cellular state, with therapeutic strategies aimed at restoring beneficial microglial programs while limiting chronic pathogenic activation.

#### 5.1.4 The role of microglia and macrophages in amyotrophic lateral sclerosis

ALS is a neurodegenerative disease characterized by moderate and progressive functional impairment as well as the loss of motor neurons (75). Neuroinflammation is a pathological feature of ALS, and its typical manifestation is an increase in reactive microglia and astrocytes in the CNS, as well as activation and infiltration of peripheral monocytes and lymphocytes into the CNS (76). The pathogenesis of ALS is usually related to the pathological aggregation of superoxide dismutase 1 (SOD1), fusion of FUS in sarcoma, or TAR DNA-binding protein TDP-43 in oligodendrocytes and astrocytes (77, 78). Multiple gene mutations expressed in neurons and microglia, such as *TARDBP, SOD1*, etc., cause ALS by disrupting autophagy, enhancing the inflammatory pathways of microglia and astrocytes, and promoting the death of motor neurons (79). In the transgenic animal models of ALS, in the early stages of the disease, activated microglia and astrocytes have anti-inflammatory effects and can protect motor neurons; however, as the disease progresses to the late stage, misfolded proteins will promote the release of injury-related molecular pattern signals, thereby activating the pro-inflammatory glial response chain, leading to accelerated damage of motor neurons (76). Thus, identifying the signaling pathways between motor neurons and glial cells is an important priority direction for developing new therapeutic strategies.

Schwann cells are one of the most thoroughly studied glial cells in the peripheral nervous system (PNS) (80). Schwann cells are typically classified as myelinating Schwann cells, non-myelinating Schwann cells, or satellite glia. Non-myelinating Schwann cells are further categorized as Remak Schwann cells or terminal Schwann cells (81). Studies have shown that ALS patients have demyelination and remyelination disorders, suggesting that Schwann cells may be functionally impaired due to the disease (82). The loss of myelinated Schwann cells reduces the overall support for motor neuron axons, thereby exacerbating axon degeneration. Alternatively, the stress induced by mutant SOD1 in Schwann cells increases oxidative stress and mitochondrial dysfunction, ultimately leading to cell death. Even before death, myelinated Schwann cells in the SOD1-G93A model exhibit functional abnormalities, including altered gene expression, reduced axonal metabolic support, and an failure to efficiently clear the remnants of degenerating axons (83). This dysfunction further weakens the motor neuron-Schwann cell unit and accelerates neurodegenerative changes.

### 5.2 The role of microglia in cerebrovascular diseases

Microglia are among the first cells in the brain to respond to cerebral ischemia and vascular injury within minutes (84). Following ischemic stroke (IS), microglia rapidly undergo morphological and functional remodeling, including process retraction, increased migration, and transition from homeostatic surveillance states toward injury-responsive states (85). Although microglial activation has historically been described using the classical M1/M2 polarization framework, increasing evidence from transcriptomic and functional studies indicates that microglial responses after CNS injury are highly heterogeneous, dynamic, and context-dependent. Rather than existing as discrete M1 or M2 populations, activated microglia exhibit a continuum of functional states, including injury-responsive, inflammatory, phagocytic, immunoregulatory, and reparative states, which are shaped by injury severity, disease stage, metabolic conditions, and local inflammatory cues.

During the early phase of ischemic stroke, injury-responsive microglia rapidly sense damage-associated molecular signals released from injured neurons, vascular cells, and extracellular matrix components, initiating adaptive immune responses and coordinating tissue surveillance. A subset of these cells may acquire an inflammatory microglial state, characterized by increased production of neuroactive and inflammatory mediators, including excitatory amino acids (such as glutamate), proteases, nitric oxide, and cytokines such as IL-6, IL-1β, IFN-γ, TNF-α, IL-15, IL-18, and IL-23, thereby contributing to secondary neuronal injury and inflammatory amplification (86, 87). However, inflammatory activation is not exclusively detrimental, as it is also required for early defense responses, debris processing, and immune coordination after ischemic injury.

Concurrently, activated microglia can adopt a phagocytic state, characterized by enhanced recognition and clearance of apoptotic cells, myelin debris, and damaged tissue components through pathways involving phagocytic receptors and lysosomal programs. This function contributes to tissue remodeling and resolution of inflammation following ischemic injury (88). In later stages of recovery, subsets of microglia may transition toward reparative and immunoregulatory states, characterized by the release of neurotrophic factors, regulation of inflammatory resolution, support of vascular repair, and restoration of tissue homeostasis. Therefore, the functional consequences of microglial activation depend not simply on the degree of inflammation but on the balance among injury sensing, inflammatory signaling, phagocytic capacity, metabolic adaptation, and repair-associated programs.

Similarly, therapeutic strategies targeting microglia after ischemic injury should not aim at simply converting a detrimental phenotype into a beneficial one, but rather at promoting protective functional states while maintaining essential immune surveillance and tissue maintenance functions. The substantial diversity and plasticity of microglia reflect their specialized role as resident macrophages of the central nervous system (89).

In cerebral small vessel disease (CSVD), cerebral microvascular dysfunction results in chronic white matter ischemic injury accompanied by persistent microglial activation (90). Under these conditions, microglia may progressively acquire inflammatory and disease-associated characteristics, accompanied by altered autophagic activity, metabolic dysregulation, and impaired cellular homeostasis (91, 92). Thus, defining microglial responses according to dynamic functional states rather than simplified polarization categories is essential for understanding disease progression and developing precise therapeutic interventions.

**Figure 2:**
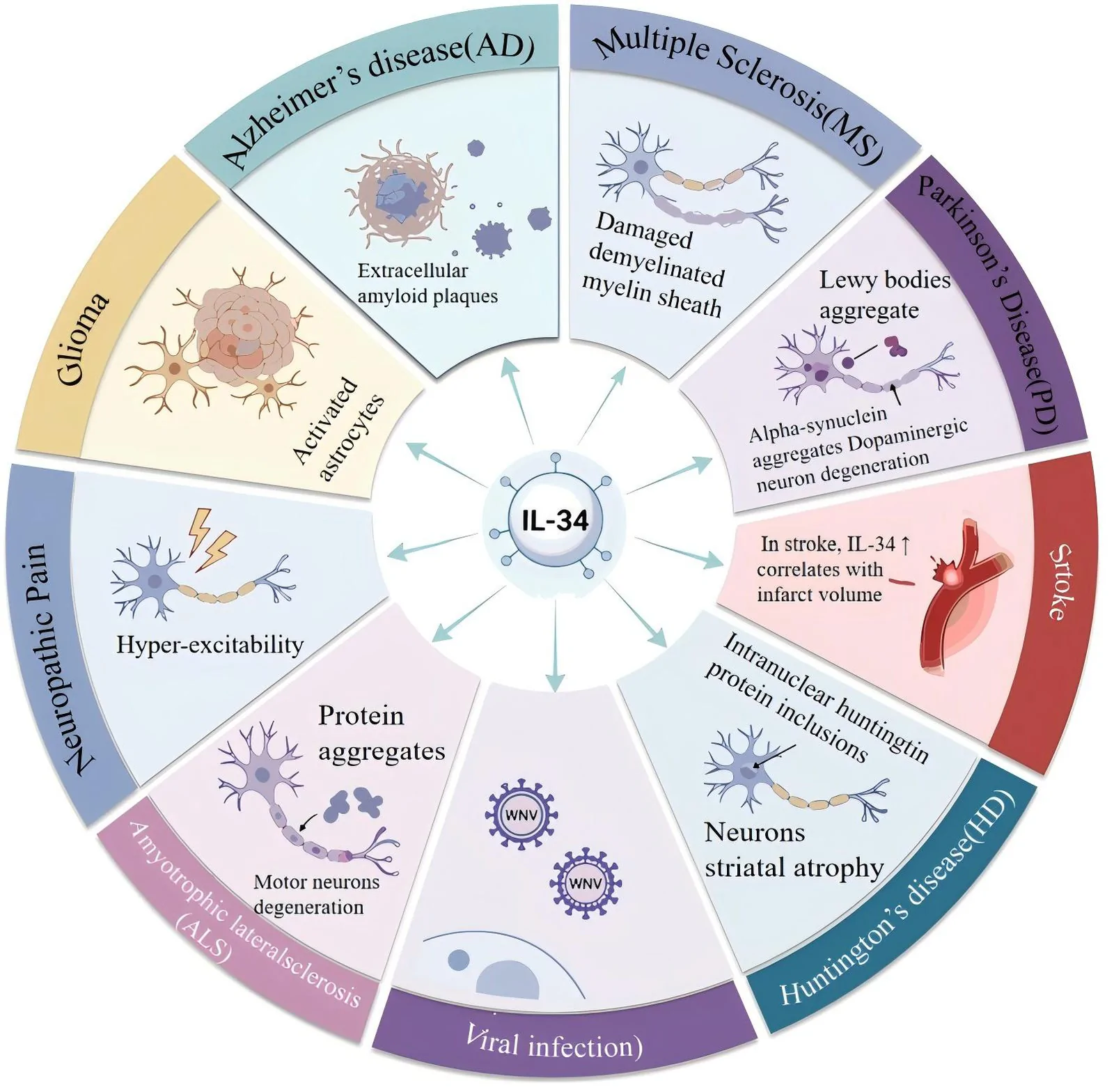
Disease contexts associated with IL-34 dysregulation in neurological disorders. IL-34 has been implicated in multiple neurological diseases; however, the strength and nature of evidence vary substantially among disease contexts. Evidence supporting IL-34 involvement ranges from human observations and disease models to *in vitro* studies and mechanistic inference. The proposed roles of IL-34 include modulation of microglial homeostasis, inflammatory responses, myeloid-cell adaptation, and tissue repair. Importantly, associations shown in this figure should not be interpreted as equivalent levels of mechanistic validation. Diseases with limited direct CNS evidence are included as emerging or hypothesis-generating contexts requiring further investigation.

The TREM2 receptor represents an important regulator of microglial adaptation during cerebral hemorrhage and ischemic injury. In mouse models, adenovirus (AAV)- and lentivirus-mediated TREM2 overexpression in mouse cells and BV2 microglia demonstrated that enhanced TREM2 signaling suppresses excessive inflammatory responses, reduces apoptosis, and improves neurological outcomes following cerebral hemorrhage (93). These findings suggest that TREM2 promotes protective microglial functions by coordinating inflammatory regulation, phagocytic activity, lipid metabolism, and cellular adaptation (**Figure 2**).

## 6 IL-34-regulated microglia and myeloid cell biology

### 6.1 Macrophage and microglial plasticity: the paradigm of myeloid reprogramming

Macrophages and microglia exhibit remarkable functional plasticity, enabling them to dynamically adapt to tissue-specific microenvironments and changing inflammatory cues. Circulating monocytes differentiate into macrophages after entering peripheral tissues, whereas microglia originate from yolk sac-derived erythromyeloid progenitors and establish a long-lived, self-renewing population within the CNS (47, 94). Despite their distinct developmental origins, both cell types continuously remodel their transcriptional, metabolic, and functional programs in response to local environmental signals. This adaptive process, commonly referred to as myeloid reprogramming, enables macrophages and microglia to coordinate immune surveillance, pathogen clearance, tissue repair, and the restoration of tissue homeostasis according to contextual demands rather than adopting fixed activation states.

Historically, macrophage and microglial activation has been described using the classical M1/M2 polarization model, in which M1 cells were considered predominantly pro-inflammatory whereas M2 cells were regarded as anti-inflammatory and tissue reparative (95). However, advances in single-cell RNA sequencing, spatial transcriptomics, and multi-omics analyses have demonstrated that this binary classification substantially oversimplifies the complexity of myeloid biology. Instead, macrophages and microglia occupy a continuum of dynamic and context-dependent functional states, including homeostatic, inflammatory, phagocytic, disease-associated, immunoregulatory, proliferative, and reparative programs, each characterized by distinct transcriptional, metabolic, and epigenetic signatures (96, 97). Importantly, no pure M1 or M2 populations expressing exclusively canonical markers have been identified *in vivo*, and individual myeloid cells frequently co-express markers traditionally assigned to both activation states. Consequently, the concept of myeloid reprogramming has gradually replaced the classical polarization paradigm, emphasizing reversible transitions among multiple functional states instructed by tissue-derived environmental cues rather than a simple switch between two opposing phenotypes (98).

### 6.2 IL-34 and potential roles in immunometabolic reprogramming of myeloid cells

Emerging evidence indicates that IL-34 functions not only as a trophic factor for macrophages and microglia but also as a tissue-derived instructive signal that orchestrates myeloid-cell reprogramming through coordinated regulation of transcriptional, metabolic, and epigenetic networks (99, 100). Epigenetic regulation—through stable yet reversible chromatin modifications such as DNA methylation, histone marks, and altered chromatin accessibility—both enables and stabilizes this reprogramming by recording environmental influences and shaping how microglia respond to future stimuli (101, 102).

Evidence from peripheral tissues supports a direct role for IL-34 in macrophage reprogramming. During gastrointestinal graft-versus-host disease (GI-GVHD), intestinal epithelial cell-derived IL-34 functions as a local niche signal that reprograms infiltrating macrophages toward an APOE-dependent tolerogenic phenotype, thereby suppressing intestinal inflammation and maintaining epithelial barrier integrity. Conversely, loss of recipient-derived IL-34 promotes inflammatory macrophage activation, increases epithelial injury, and enhances the accumulation of pathogenic GM-CSF-producing CD4^+^ T cells, whereas recombinant IL-34 restores macrophage reprogramming and significantly attenuates GVHD severity and mortality (103). These findings demonstrate that IL-34 actively instructs tissue-specific macrophage adaptation rather than merely supporting macrophage survival. Although these studies provide important mechanistic insights into IL-34-dependent myeloid plasticity, they were primarily derived from peripheral inflammatory diseases and should not be directly interpreted as evidence for identical mechanisms in CNS-resident microglia. Instead, they provide conceptual frameworks suggesting that IL-34 may function as a tissue-derived instructive signal capable of shaping myeloid-cell states in a context-dependent manner.

Comparable mechanisms have been described in the CNS. A recently established cytokine-driven reprogramming system showed that PMA-differentiated THP-1 macrophages exposed to IL-4, CSF-1, IL-34, and TGF-β acquire transcriptional and metabolic characteristics partially resembling disease-associated microglia and lipid-associated macrophages (DLAMs) observed in AD. Within this cocktail, IL-34 and CSF-1 primarily provide brain-specific CSF-1R signals that direct macrophages toward a microglia-like identity, whereas IL-4 promotes reparative transcriptional programs (104, 105). Complementary findings from patient-derived induced microglia-like cells further demonstrate that IL-34, in conjunction with GM-CSF, efficiently drives monocyte-to-microglia conversion, yielding TMEM119-positive cells with heightened inflammatory and phagocytic responses, particularly in cells derived from individuals with AD (106). Functionally, the cells reprogrammed by cytokines exhibit reduced migration, phagocytosis, and lysosomal protein hydrolysis capabilities towards Aβ, myelin, and apoptotic cells, but enhanced glycolysis and mitochondrial metabolism, increased lipid droplet accumulation, and increased APOE secretion (107).

Recent advances have further extended this concept by proposing a Genetic-Environmental Programming model of microglial identity, in which lineage-determining transcription factors and epigenetic landscapes are continuously shaped by brain-derived trophic cues and peripheral environmental signals. Within this framework, the CSF-1/IL-34–CSF-1R axis represents a prototypical environmental programming pathway. PU.1 and IRF8 establish permissive chromatin accessibility and enhancer priming during early microglial development, whereas neuron-derived IL-34 and glia-derived CSF-1 activate CSF-1R to induce downstream transcription factors such as AP-1 and EGR1, which cooperate with lineage-defining transcription factors to stabilize microglial gene-expression programs. This coordinated signaling contributes to region-specific transcriptional identities and developmental transitions between transient activated microglial states and mature homeostatic microglia through dynamic remodeling of enhancer landscapes, including H3K27ac-associated chromatin modifications (100, 106). Accordingly, IL-34 may participate in the regulation of chromatin-associated programs through sustained CSF-1R-dependent transcriptional and metabolic signaling.

Collectively, current evidence indicates that IL-34 is an important regulator of myeloid-cell reprogramming by integrating tissue-derived signals with transcriptional and metabolic adaptation. Although IL-34 has been implicated in shaping glycolytic activity, mitochondrial metabolism, lipid handling, and microglial identity, direct evidence linking IL-34 to specific epigenetic modifications remains lacking. Future studies combining metabolic flux analysis with chromatin accessibility and histone-modification profiling will be critical for determining whether IL-34 establishes a mechanistic link between immunometabolic remodeling and epigenetic regulation, thereby stabilizing context-dependent microglial states during CNS homeostasis and disease.

## 7 Research on IL-34 in neurological diseases

### 7.1 The role of IL-34 in AD

AD is characterized by extracellular Aβ deposition and intracellular neurofibrillary tangles, with microglial dysfunction increasingly recognized as a key driver of disease progression (108). Microglia normally restrain pathology by clearing soluble and oligomeric Aβ species, thereby delaying plaque accumulation (109). Within this context, IL-34 has emerged as an important regulator of microglial biology; however, accumulating evidence indicates that its effects in AD are not intrinsically neuroprotective or detrimental, but instead depend on disease stage, receptor availability, microglial functional state, and the surrounding inflammatory environment.

During the early or compensatory stages of AD, when microglial homeostatic programs remain relatively preserved, IL-34 may contribute to protective responses by supporting microglial survival, proliferation, and functional adaptation. In several rodent models, IL-34 administration enhanced microglial antioxidant capacity, proteolytic activity, and Aβ clearance, accompanied by improved cognitive performance (110).

However, as AD progresses and chronic neuroinflammation, lipid dysregulation, and microglial dysfunction become established, persistent IL-34 signaling may no longer promote beneficial adaptation and may instead contribute to maladaptive inflammatory responses. In the 3x-Tg AD mouse model, chronic IL-34 exposure aggravated Aβ accumulation, tau hyperphosphorylation, and neuroinflammation, accompanied by increased expression of inflammatory cytokines including TNF-α, IL-1β, and IL-6 (111). These findings suggest that excessive or prolonged IL-34 activity may expand or maintain activated microglial populations that are unable to efficiently restore homeostasis. Importantly, the detrimental effects observed in chronic models do not indicate that IL-34 is inherently pathogenic; rather, they reflect a pathological context in which inflammatory signaling, altered metabolism, and impaired microglial plasticity may determine the final outcome of IL-34 stimulation.

The apparently divergent effects of IL-34 can be better understood by considering the coordinated contribution of its two major receptors, CSF-1R and TREM2. Rather than representing opposing pathways in which CSF-1R mediates inflammation and TREM2 mediates protection, both receptors participate in regulating microglial survival, adaptation, and functional specialization. Through CSF-1R, IL-34 activates PI3K/AKT, ERK/MAPK, and JAK/STAT pathways that maintain microglial survival, proliferation, differentiation, and metabolic fitness (17).

Recent work further identified TREM2 as an additional functional receptor for IL-34. Structural and functional analyses demonstrated that IL-34 binds TREM2 through a distinct basic interface and activates DAP12/SYK-associated signaling, resulting in suppression of NLRP3 inflammasome activation and attenuation of Aβ-induced inflammatory responses (18). *In vivo*, IL-34 administration reduced oxidative stress and cognitive impairment in SAMP8 mice, supporting a protective role of IL-34/TREM2 signaling under conditions where microglial phagocytic and metabolic functions remain responsive (18). Importantly, TREM2 signaling may determine whether IL-34-driven microglial expansion results in beneficial adaptation or dysfunctional activation. In early AD, preserved TREM2 activity may facilitate Aβ sensing, lipid metabolism, and phagocytic clearance, whereas in advanced disease characterized by chronic inflammation, lipid dysregulation, or impaired TREM2/APOE signaling, IL-34-mediated expansion of microglia may instead contribute to persistent inflammatory activation. Therefore, CSF-1R and TREM2 should not be viewed as protective and inflammatory pathways, respectively; rather, their relative activity and interaction with the local microenvironment determine the functional outcome of IL-34 signaling.

**Figure 3:**
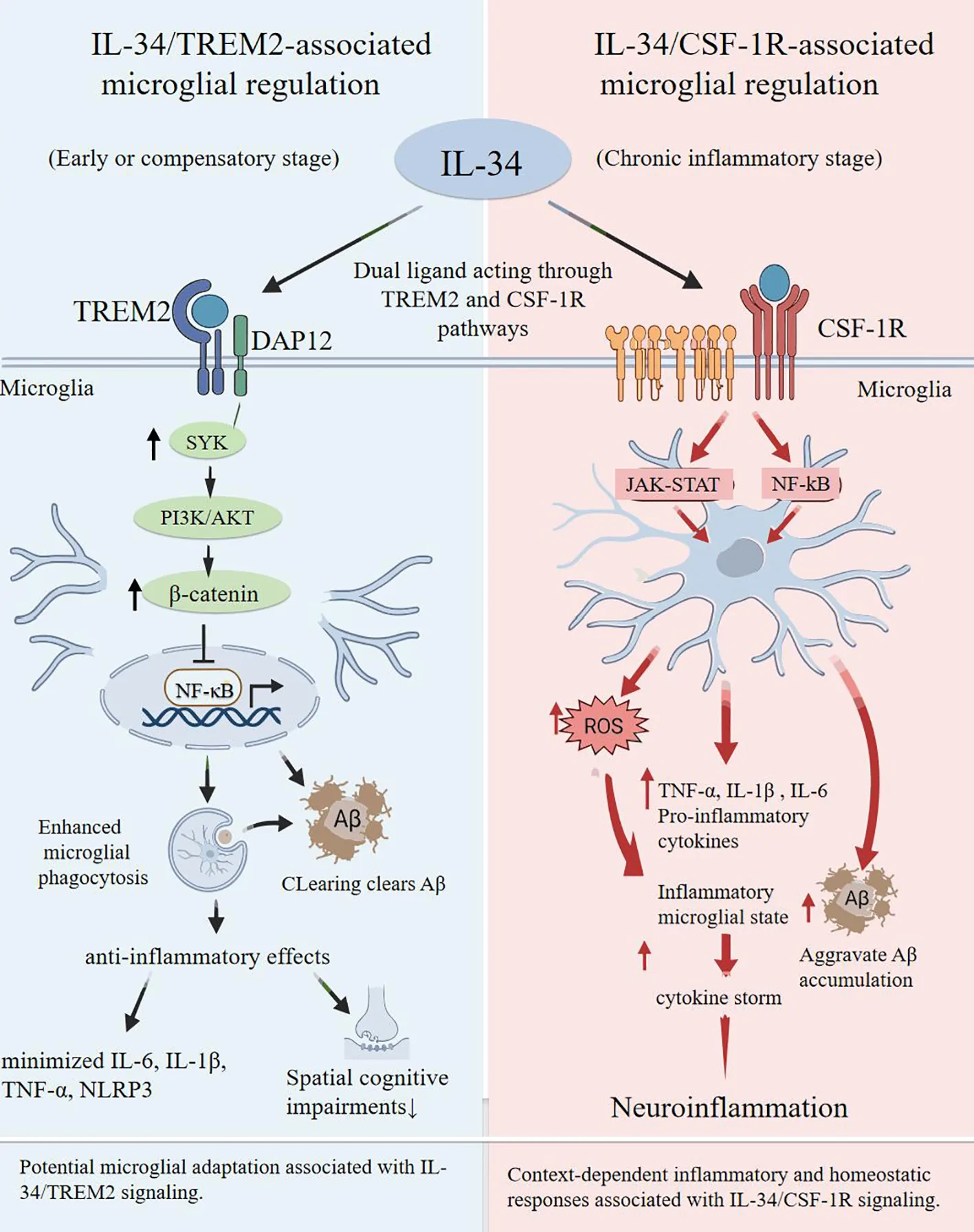
Context-dependent regulation of microglial responses by IL-34 signaling in AD. IL-34 acts as a dual ligand that regulates microglial function through both TREM2- and CSF-1R-associated signaling pathways. On the TREM2 side, IL-34 binding may activate DAP12-associated SYK signaling and downstream PI3K/AKT–β-catenin pathways, which can enhance microglial phagocytic activity, promote Aβ clearance, and attenuate inflammatory responses under specific disease conditions. On the CSF-1R side, IL-34 contributes to microglial survival, maintenance, proliferation, and functional adaptation through downstream pathways including JAK–STAT and NF-κB signaling. However, excessive or dysregulated CSF-1R activation in an inflammatory microenvironment may promote oxidative stress, pro-inflammatory cytokine production, and impaired Aβ homeostasis.

The research on human brain samples also indicates that the effect of IL-34 may not have the obvious protective effect as shown in animal experiments. Instead, it shows a stronger pro-inflammatory effect and may weaken the clearance of Aβ. Walker et al. analyzed post-mortem human brain tissues and human brain-derived microglial cells from the elderly and found that in the cortical regions affected by AD, the transcriptional levels of CSF-1R and CSF-1 increased, while the expression of IL-34 decreased in the inferior temporal gyrus region. This suggests that the ligand-receptor balance within this pathway has been disrupted in specific regions. At the same time, transcriptomic analysis indicated that IL-34 and CSF-1 induced highly similar responses in human microglial cells, and both of these ligands promoted inflammatory-dominated transcriptional programs rather than obvious reparative phenotypes (112). These findings indicate that observations from human tissues may differ from experimental animal models because of differences in disease duration, inflammatory environment, receptor expression, and microglial state (**Figure 3**).

Beyond resident microglia, IL-34 differentially affects peripheral MDMs. *In vitro*, IL-34 impairs monocyte differentiation and reduces Aβ uptake (113), whereas in the CNS parenchyma, host-derived IL-34 is essential for the survival, MHCII^+^ polarization, and functional adaptation of engrafted MDMs. In the absence of IL-34, MDMs lose their immunologically active identity and adopt a quiescent, microglia-like phenotype (12). These findings further emphasize that IL-34 effects depend on cellular origin, tissue niche, and differentiation status, rather than producing a uniform response across myeloid populations.

Clinical and genetic evidence also reflects this complexity. A multicenter cohort study reported progressive increases in IL-34 levels from subjective cognitive impairment (SCI) to mild cognitive impairment (MCI) and AD, with higher IL-34 concentrations associated with poorer cognitive performance and detectable changes across saliva, plasma, and cerebrospinal fluid (CSF) samples (114). These associations may reflect increased peripheral immune activation, blood–brain barrier dysfunction, or altered CNS immune signaling rather than a direct pathogenic effect of IL-34 itself (115). Multi-omics and MR analyses further suggest that higher IL-34 levels are causally associated with increased AD risk, and that the IL-34/TREM2 signaling axis is upregulated in AD cortex (116–118), supporting a role for IL-34 as both a marker and potential regulator of disease-associated immune remodeling.

Taken together, current evidence supports a stage- and context-dependent model of IL-34 function in AD. During early disease, IL-34 may preserve microglial fitness, enhance phagocytic capacity, and support Aβ clearance, whereas prolonged signaling in an inflammatory and metabolically disturbed environment may contribute to maladaptive microglial activation and neuroinflammation. Therefore, IL-34 should not be classified as simply neuroprotective or pathogenic; rather, its effects are determined by the dynamic interaction between disease stage, CSF-1R/TREM2 signaling balance, microglial functional state, and the local inflammatory milieu.

### 7.2 The role of IL-34 in PD

PD is increasingly recognized as a multisystem neurodegenerative disorder involving not only the CNS but also the enteric and autonomic nervous systems and peripheral immune compartments, with a clinical spectrum spanning motor and non-motor symptoms (119). Neuroinflammation is consistently observed in PD, although whether it is a primary driver or a disease-amplifying consequence remains unresolved (120). Among immune cells, microglia are strongly implicated in nigrostriatal degeneration, with activated microglia exhibiting altered inflammatory, metabolic, and phagocytic programs accompanied by increased expression of cytokines including IL-1α, IL-1β, TNF-α, IL-6, TGF-β, and IFN-γ (121, 122). Within this complex inflammatory network, IL-34 has emerged as a potential regulator of microglial responsiveness, although its precise temporal and causal position remains to be fully established.

Experimental evidence suggests that IL-34 can modulate the magnitude of microglial inflammatory responses under pathological conditions. Kim et al. (2017) demonstrated that systemic LPS challenge in a PD mouse model induced microglial and astrocytic activation together with increased expression of inflammatory mediators, including TNF-α, IL-1β, and IL-6. *In vitro*, IL-34 pretreatment of BV2 microglia enhanced LPS-induced inflammatory responses, increasing TNF-α production and nitric oxide generation through CSF-1R-dependent activation of p38 MAPK and NF-κB signaling (123). These findings indicate that IL-34 can increase microglial sensitivity to inflammatory stimulation and amplify downstream inflammatory outputs. However, this evidence does not establish IL-34 as an initiating cytokine or a primary driver of inflammation, because IL-34 expression itself may be regulated by inflammatory signals and was examined mainly in the context of pre-existing immune activation.

Consistent with this interpretation, IL-34 should be considered as a component of a reciprocal inflammatory network rather than a unidirectional upstream regulator. In PD models, IL-34 may participate in feed-forward interactions in which inflammatory stimuli enhance IL-34/CSF-1R signaling, while IL-34 subsequently reinforces microglial activation and inflammatory mediator production. Supporting this concept, inhibition of matrix metalloproteinase-8 (MMP-8) reduced IL-34-associated inflammatory responses, attenuated astrocyte activation in the substantia nigra and hippocampus, and improved motor deficits in PD mice (75). Nevertheless, whether increased IL-34 represents a compensatory response, a disease-amplifying signal, or both likely depends on disease stage, inflammatory context, and microglial functional state.

Overall, current evidence supports a role for IL-34 as a context-dependent modulator of neuroinflammation in PD. Rather than acting as a proven priming factor that initiates inflammatory cascades, IL-34 may function as an amplifier that shapes the intensity and persistence of microglial responses after inflammatory activation. Future studies using temporal profiling, cell-type-specific IL-34/CSF-1R manipulation, and longitudinal human samples will be required to determine whether IL-34 acts primarily as a driver, consequence, or feedback regulator during PD progression.

### 7.3 The role of IL-34 in MS

MS is a chronic autoimmune demyelinating disorder in which dysregulated interactions between adaptive and innate immunity drive CNS inflammation and tissue damage (124). IL-34 has recently emerged as a potential mediator at this interface, though its precise role in MS pathogenesis remains incompletely defined.

Mechanistically, IL-34 primarily functions through CSF-1R signaling to regulate the survival, differentiation, and functional adaptation of macrophage-lineage cells. Studies in peripheral immune systems have shown that IL-34 can be produced by regulatory immune cells, including CD4^+^FOXP3^+^ regulatory T cells, and may regulatory or homeostatic macrophage responses and enhance regulatory T-cell functions through CSF-1R-dependent mechanisms (125). These findings suggest that IL-34 may contribute to immune homeostasis by restraining excessive myeloid activation. However, whether these peripheral immunoregulatory mechanisms directly operate within the CNS during MS remains unclear. Although defects in regulatory T-cell function, including impaired suppressive activity of CD8^+^ regulatory T cells, are well documented in MS patients (126) , direct evidence linking reduced IL-34 production to impaired immune regulation and MS progression is currently lacking.

Evidence from experimental autoimmune encephalomyelitis (EAE), the most widely used animal model of MS, further suggests that IL-34 does not function as a dominant driver of acute autoimmune neuroinflammation. Hwang et al. demonstrated that neutralization of IL-34 did not significantly affect disease onset or severity, whereas inhibition of CSF-1 or CSF-1R markedly reduced disease progression and demyelinating pathology (127). These findings indicate that although both IL-34 and CSF-1 signal through CSF-1R, their functional contributions during neuroinflammation are not equivalent. CSF-1 may preferentially support inflammatory myeloid expansion during active autoimmune responses, whereas IL-34 may play a more prominent role in maintaining microglial identity and tissue homeostasis under steady-state or repair-associated conditions. Nevertheless, because CSF-1R signaling is essential for microglial survival and macrophage biology, broad inhibition of this pathway may affect both pathogenic and protective myeloid functions.

Clinical observations provide additional but still limited evidence. Elevated IL-34 levels have been detected in the cerebrospinal fluid of patients with MS compared with healthy controls (128). However, increased IL-34 should not be interpreted as direct evidence of pathogenic activity. Instead, elevated IL-34 may represent a disease-associated response reflecting altered CNS immune regulation, microglial activation, or an attempt to restore tissue homeostasis. Neuron-, glia-, and endothelial-derived IL-34 may contribute to maintaining microglial survival, regulating myeloid-cell adaptation, and supporting CNS barrier function, although these proposed protective effects require further validation in MS-specific models.

Collectively, current evidence suggests that IL-34 is unlikely to act as a primary pathogenic driver in MS. Rather, IL-34 appears to function as a context-dependent regulator of myeloid-cell homeostasis, with potential roles in limiting excessive inflammation and supporting tissue maintenance. Future studies using conditional IL-34 manipulation in CNS-resident microglia and infiltrating myeloid populations are required to determine whether IL-34-mediated signaling represents a protective response, a disease-associated adaptation, or a context-dependent contributor to MS pathology.

### 7.4 The role of IL-34 in cerebrovascular diseases

Ischemic stroke (IS) triggers a dynamic neuroimmune response in which resident microglia and infiltrating monocytes coordinate both acute tissue injury and subsequent repair. Increasing evidence suggests that IL-34 is involved throughout this process, although its biological functions vary according to disease stage. During the acute phase of IS, circulating IL-34 levels are significantly elevated and positively correlate with infarct volume and neurological deficit, supporting its potential value as an independent prognostic biomarker (129).

In the chronic stage, persistent dysregulation of IL-34 appears to be associated with unfavorable neurological outcomes. Elevated plasma IL-34 levels have been linked to an increased risk of Chronic post-stroke cognitive impairment (PSCI) and poorer cognitive performance (130). These observations suggest that while acute IL-34 responses may initially support tissue repair, prolonged or excessive IL-34 signaling contributes to maladaptive neuroimmune remodeling and cognitive decline.

Beyond overt stroke, IL-34 is also implicated in chronic cerebrovascular disorders, particularly cerebral small vessel disease (CSVD). Higher circulating IL-34 levels correlate positively with the triglyceride-glucose (TyG) index and insulin resistance, both recognized risk factors for vascular cognitive impairment (VCI) (131). In contrast, patients with established vascular dementia (VaD) exhibit reduced serum IL-34 levels compared with cognitively normal individuals, and lower IL-34 concentrations are associated with more severe cognitive impairment (132). These apparently divergent findings likely reflect stage-dependent changes in IL-34 biology during disease progression, with IL-34 contributing to early inflammatory and metabolic responses but becoming insufficient to support microglial homeostasis and neurovascular integrity in advanced disease.

Taken together, current evidence identifies IL-34 as a context-dependent regulator across the spectrum of cerebrovascular diseases. Rather than exerting uniformly detrimental or protective effects, IL-34 appears to influence inflammatory activation, microglial homeostasis, tissue remodeling, and cognitive outcomes in a stage-specific manner. Consequently, therapeutic modulation of the IL-34/CSF-1R axis will likely require precise consideration of disease stage and the underlying neuroimmune environment to maximize benefit while minimizing unintended effects.

### 7.5 Other neural system diseases

In addition to common neurodegenerative disorders, the IL-34/CSF-1R signaling axis shows pronounced pleiotropy across a range of CNS and PNS conditions, with outcomes largely determined by the initiating stressor, the cellular source of IL-34, and the effector myeloid population that is recruited or expanded. Neuronal IL-34 can be induced by diverse stress signals, including DNA damage and NMDA receptor-mediated excitotoxicity. In this setting, IL-34 primarily functions as a neuron-derived cue that increases microglial abundance and responsiveness, which can be beneficial for clearance and repair but may also facilitate neurotoxicity when paired with pro-inflammatory programs. In Huntington’s disease (HD), mutant huntingtin protein (mHTT) activates IKKβ signaling, which promotes neuronal IL-34 expression and accelerates microglia-driven striatal degeneration (133). A similar logic applies to post-infectious synaptic pathology. In a murine model of attenuated West Nile virus (WNV) infection that recapitulates cognitive sequelae after neuroinvasive disease, persistent hippocampal microglial activation occurred without overt neuronal loss but was associated with spatial memory impairment. Mechanistically, infected neurons activated the classical complement cascade (C1q to C3), enabling microglia to engulf presynaptic terminals in CA3 through C3a receptor signaling. Mice lacking IL-34, which exhibit reduced microglial density due to impaired CSF-1R ligand support, were protected from virus-induced synaptic elimination; C3 or C3a receptor deficiency similarly prevented synapse loss (134). Together, these findings support an IL-34-CSF-1R-complement axis in which IL-34-supported microglial expansion provides the cellular substrate for complement-tagged synapse removal, offering a mechanistic explanation for long-term cognitive impairment after neurotropic viral infection.

**Figure 4:**
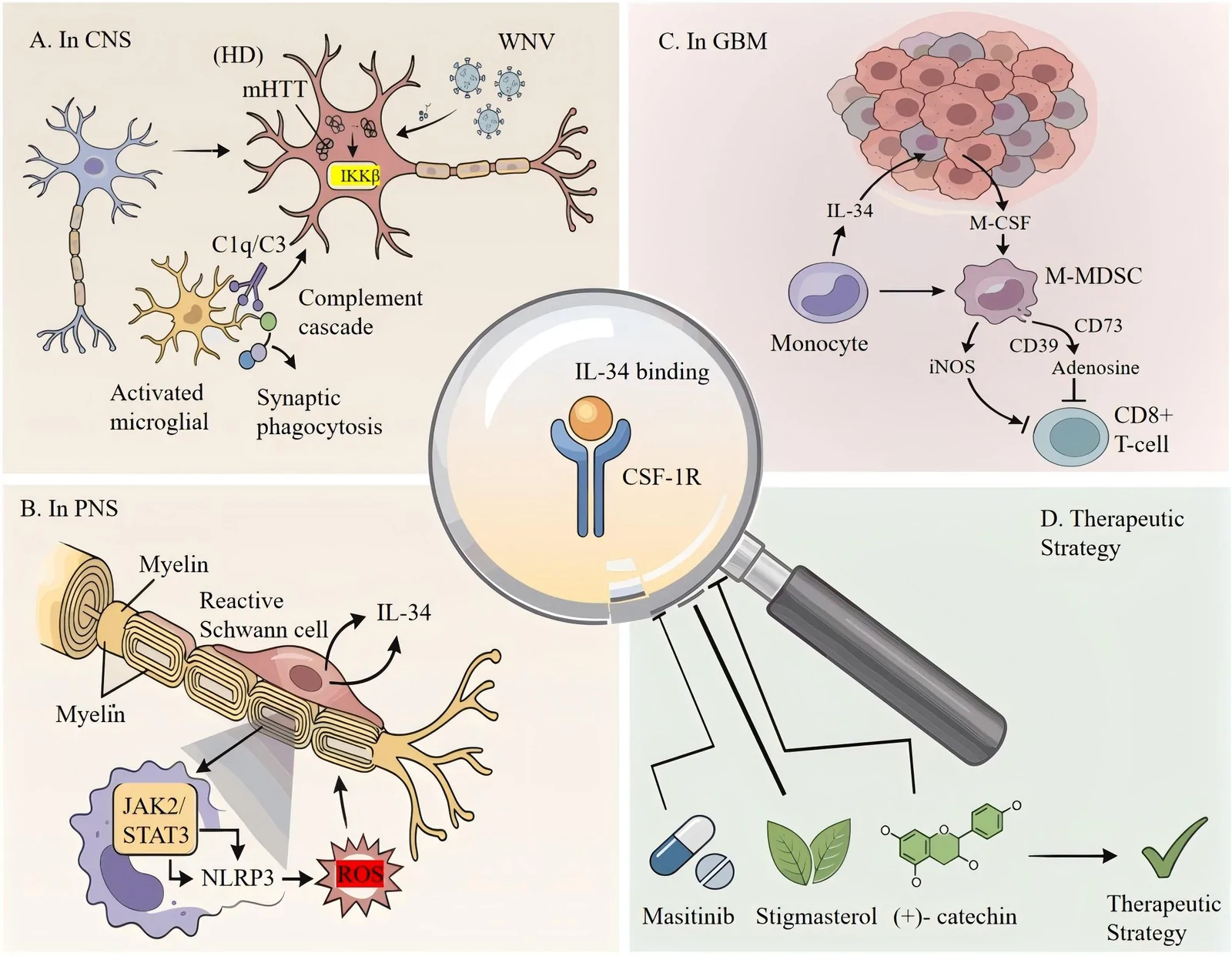
Overview of IL-34/CSF-1R signaling across the CNS and PNS and its therapeutic implications **(A)** IL-34 contributes to microglial expansion and complement-associated synaptic remodeling **(B)** The IL-34 secreted by Schwann cells promotes oxidative stress and enhances the inflammatory response of the peripheral nervous system. **(C)** However, in glioblastoma, tumor-derived IL-34 supports the expansion of immunosuppressive myeloid cells that inhibit cytotoxic T-cell activity. **(D)** Several pharmacological agents, including masitinib, stigmasterol, and (+)-catechin, target components of the IL-34/CSF-1R axis and represent potential therapeutic strategies.

In the peripheral nervous system (PNS), Schwann cells (SCs) represent a major local source of IL-34 (135). In Amyotrophic lateral sclerosis (ALS), IL-34 is markedly upregulated during motor axon degeneration and is produced predominantly by a subset of denervated Schwann cells (GFAP^+^/isolectin^+^) rather than by myelin-laden phagocytic Schwann cells. As a cognate ligand for CSF-1R, Schwann cell-derived IL-34 is positioned to act in a paracrine manner on endoneurial CSF-1R^+^ monocytes/macrophages that accumulate around reactive SCs, thereby promoting myeloid expansion/activation and sustaining local neuroinflammation. The study also notes occasional IL-34 signal within axons, consistent with an additional neuronal/axonal source that could further reinforce CSF-1R signaling along degenerating motor pathways. Therapeutically, blocking CSF-1R (together with c-Kit) using masitinib reduces peripheral immune infiltration and Schwann cell reactivity, supporting IL-34/CSF-1R signaling as part of an amplifying inflammatory loop in ALS distal axonopathy (135). Neuropathic pain models further implicate IL-34 in non-neuronal inflammatory circuits that drive sensory hypersensitivity. Single-cell transcriptomics of dorsal root ganglia after nerve injury identified increased IL-34 and CSF-1R expression in activated stromal and immune populations. Experimental work suggests that IL-34 produced in injured ganglia promotes macrophage proliferation, migration, and inflammatory mediator release through ERK, NF-κB, and JAK2/STAT3 signaling, with downstream amplification via the NLRP3 inflammasome and reactive oxygen species generation, ultimately sensitizing sensory neurons and sustaining chronic pain (136). Pharmacological interventions that reduce IL-34 production, including stigmasterol and (+)-catechin, attenuated CSF-1R-associated macrophage activation, lowered pro-inflammatory cytokines and chemokines in serum and ganglia, preserved sciatic nerve integrity, and improved pain hypersensitivity *in vivo* (137). In the chronic constriction injury (CCI) model, Schwann cells upregulate IL-34, which engages CSF-1R on infiltrating macrophages, activates JAK2/STAT3 signaling, and amplifies a Schwann cell–macrophage inflammatory loop that drives cytokine release and neuronal sensitization. Treatment with (+)-catechin suppresses Schwann cell IL-34 production, limits CSF-1R-dependent macrophage activation, and attenuates downstream JAK2/STAT3 signaling (138). These findings highlight the IL-34-CSF-1R-JAK2/STAT3 pathway as a therapeutically accessible non-neuronal circuit underlying neuropathic pain (**Figure 4**).

In contrast to inflammatory and degenerative settings, IL-34 can support immune evasion in cancer. In glioblastoma, tumor-derived IL-34 together with CSF-1 promotes differentiation of bone marrow-derived monocytes toward monocytic myeloid-derived suppressor cells, which suppress anti-tumor T-cell responses through iNOS and CD39/CD73-mediated adenosine signaling (139). This illustrates that the same ligand-receptor system can drive immunosuppression when the tumor microenvironment imposes an alternative myeloid differentiation program.

Under these conditions, IL-34 is best viewed as a local niche factor that controls the size and state of CSF-1R-expressing myeloid populations. In the CNS, it can facilitate microglial expansion and complement-enabled synaptic remodeling after infection and can exacerbate microglia-mediated neurodegeneration in HD. In the PNS, it supports a Schwann cell-macrophage inflammatory loop that sustains Wallerian degeneration and neuropathic pain. In glioma, it biases infiltrating myeloid cells toward immunosuppressive phenotypes. These context-specific effects argue for disease- and compartment-tailored modulation of the IL-34/CSF-1R signaling pathway, including CSF-1R kinase inhibitors (for example, masitinib) or compounds that suppress glial IL-34 production rather than uniform systemic inhibition.

## 8 Discussion

In the CNS, IL-34 appears to extend beyond maintaining microglial survival to shaping how resident and recruited myeloid cells interpret tissue-derived stress signals. This is particularly relevant to neurodegenerative diseases, where the effects of IL-34 are clearly not unidirectional. Under physiological conditions, IL-34 is produced mainly by neurons and supports microglial homeostasis and regional specialization, especially in forebrain circuits. In disease, however, IL-34 is more likely induced as part of a broader injury-associated program, including excitotoxic stress, proteotoxic burden, and inflammatory signaling. Thus, increased IL-34 should not be interpreted as an isolated protective response but rather as one component of a local immune-conditioning niche that simultaneously alters receptor expression, metabolic state, and transcriptional responsiveness in myeloid cells (37, 140). In AD, the role of IL-34 has been studied most thoroughly, but its mechanism is also more complex. This framework helps explain the dual role of IL-34 in AD and related disorders. IL-34 may be beneficial when it supports a microglial state still capable of effective phagocytosis, lysosomal processing, redox control, and inflammatory resolution (110). In contrast, in a tissue environment characterized by sustained TNF-α and IL-1β exposure, lipid overload, defective proteostasis, complement activation, and inflammasome priming, the same signal may instead preserve a maladaptive population of chronically activated but functionally inefficient myeloid cells. In this sense, the bidirectional effects of IL-34 are less likely to reflect time alone and more likely to reflect the quality of the myeloid state that IL-34 helps maintain (112) (141). This interpretation may also account for the discrepancy between experimental systems in which IL-34 enhances amyloid clearance and human AD tissue, where increased signaling through this axis does not necessarily restore microglial competence.

An additional issue is that IL-34 appears to participate directly in myeloid reprogramming. Evidence from macrophage systems and human microglia-like models indicates that IL-34 does not merely support cell viability but helps shape transcriptional identity, metabolic activity, and functional specialization. In CNS-relevant settings, this likely occurs through sustained CSF-1R signaling integrated with local cues such as TGF-β, lipid stress, complement activation, and neuronal damage signals. The result is not a single fixed phenotype but a shift in the threshold at which myeloid cells adopt disease-associated states. This may be particularly relevant in AD, where IL-34-containing cytokine combinations can promote microglia-like or lipid-associated programs, while *in vivo* outcomes depend on whether those cells remain competent for phagocytosis and degradation or instead become chronically activated and metabolically strained (18).

The link between IL-34 and epigenetic regulation is also likely to deserve more attention. At present, direct evidence that IL-34 controls specific chromatin marks in CNS myeloid cells remains limited, and this point should be stated cautiously. Nevertheless, the available data support a plausible model in which IL-34 contributes to epigenetic reprogramming indirectly by altering signaling and metabolism. Through CSF-1R, IL-34 engages pathways such as PI3K-AKT, MAPK, STAT, and NF-κB, all of which are known to influence enhancer activity, inflammatory gene expression, and metabolic adaptation. Because metabolic intermediates help determine the availability of substrates for chromatin modification, IL-34-driven changes in glycolysis, mitochondrial function, or lactate production could shape the epigenetic permissiveness of microglia. In this framework, IL-34 may not impose a transcriptional program on its own but may influence how microglia interpret concurrent disease signals and how stable those state transitions become over time.

This broader view may also help explain why IL-34 has different effects in cancer. IL-34 is not intrinsically protective or harmful; rather, it tends to stabilize the dominant myeloid program of the local niche. In solid tumors, including brain metastases of lung adenocarcinoma, IL-34 and its receptor network are often upregulated and are associated with disease progression, suggesting a role in shaping a tumor-supportive microenvironment. By contrast, in hematologic malignancies such as AML, IL-34 may promote differentiation through alternative receptor usage, including TREM2, thereby limiting leukemic progression. The key determinant therefore appears to be receptor context, cellular differentiation state, and the transcriptional program already active in the responding cell. A similar principle is likely to apply in the diseased brain, where IL-34 may either preserve microglial competence or prolong maladaptive immune persistence.

Taken together, the current literature supports a model in which IL-34 acts as a context-dependent regulator of myeloid cell plasticity rather than a uniformly beneficial or detrimental cytokine. Future work should focus less on bulk IL-34 abundance alone and more on the conditions under which IL-34 is induced, the receptor combinations engaged, and the metabolic and chromatin states of responding cells. This will be essential for determining whether therapeutic modulation of IL-34 should aim to enhance homeostatic signaling, interrupt pathological myeloid persistence, or selectively target specific receptor axes in different neurological diseases.
